# Misclassification of PfEH1 and PfEH2 as Epoxide Hydrolases

**DOI:** 10.1128/mBio.00004-17

**Published:** 2017-03-07

**Authors:** Michael Arand, Anne Marowsky

**Affiliations:** Institute of Pharmacology and Toxicology, University of Zurich, Zurich, Switzerland; NIAID/NIH

## LETTER

In their recent article (1), Spillman and colleagues report the two *Plasmodium falciparum* proteins PfEH1 and PfEH2 to be two novel epoxide hydrolases with an atypical active-site architecture. We strongly believe that this conclusion is wrong for the reasons detailed in the following.

Spillman et al. start from the observation that, based on sequence identity, epoxide hydrolases from the α/β hydrolase fold enzyme superfamily are the closest relatives found for PfEH1 and -2. However, the largest group of enzymes within this superfamily are esterases (2). An easy way to predict whether a novel enzyme of unknown function within this superfamily is likely an esterase or an epoxide hydrolase is to look at the catalytic nucleophile, which can easily be identified by sequence comparison (3). All epoxide hydrolases described so far have an aspartic acid as the catalytic nucleophile, while esterases, with very few exceptions, have a serine residue in this position.

**FIG 1:**
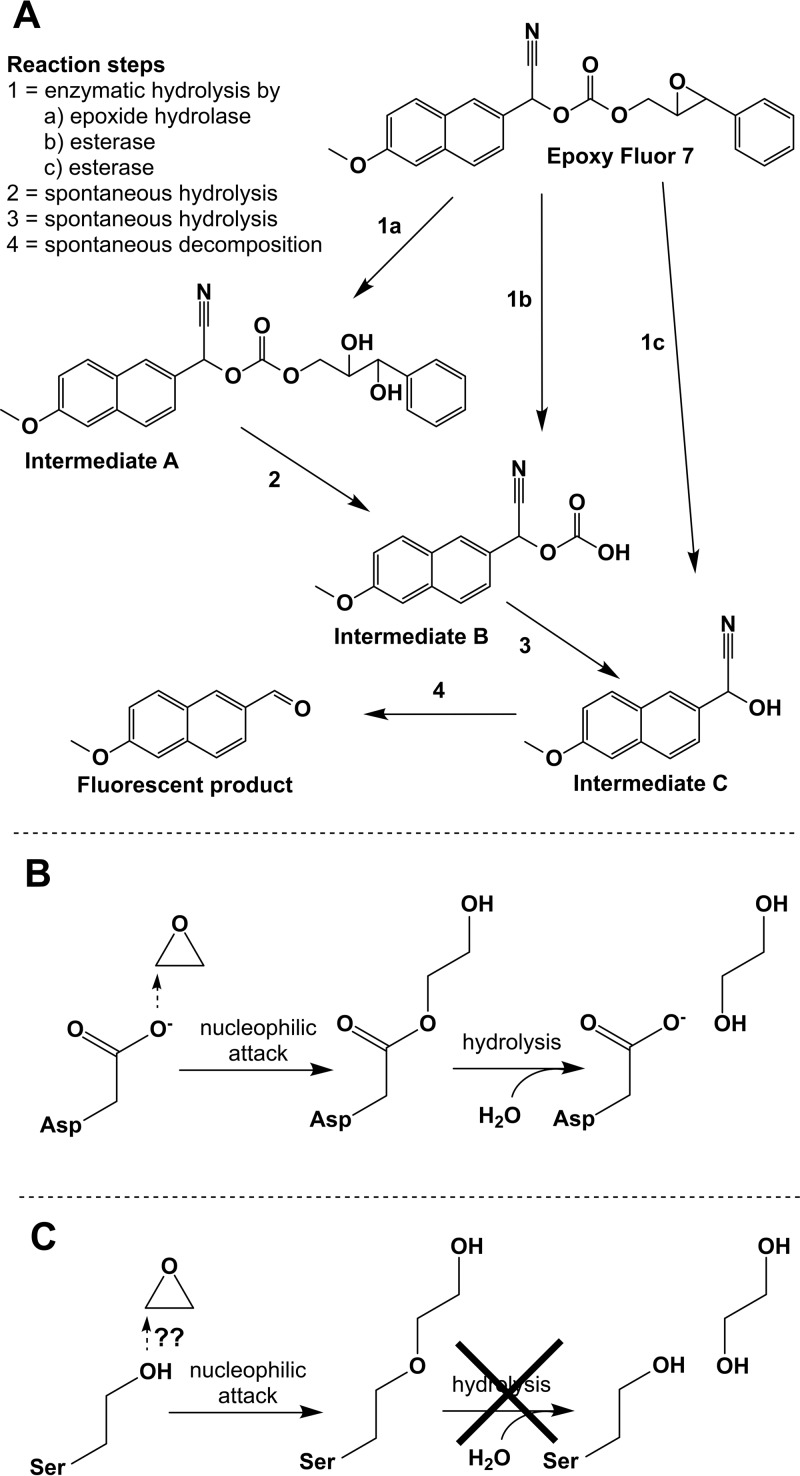
Reaction schemes for epoxide turnover. (A) Enzymatic hydrolysis pathways for epoxy fluor 7; (B) generic hydrolysis mechanism for epoxides by α/β hydrolase fold epoxide hydrolases; (C) consequence of a serine in place of the catalytic nucleophile for epoxide hydrolysis. Further explanations are given in the text.

Spillman et al., despite finding a serine at the position of the catalytic nucleophile in PfEH1 and -2, tested the activities of the purified proteins with the commercial epoxide hydrolase substrate epoxy fluor 7 (EF7) and found substantial turnover with PfEH1. Unfortunately, they neglected the fact that EF7 is also a useful substrate for many esterases (the supplier of the substrate does not indicate that). In fact, hydrolysis of the epoxide moiety of EF7 induces the subsequent spontaneous hydrolysis of two ester bonds within the resulting metabolite to finally release the fluorescent product that indicates turnover (Fig. 1A). Thus, enzymatic hydrolysis of either of these ester bonds would be equally effective in the hydrolysis of the epoxide group itself in generating the fluorophore. Thus, EF7 does not allow discrimination between epoxide hydrolases and esterases.

In addition, Spillman et al. assessed the activities of the two enzymes against specific substrates, namely, epoxyeicosatrienoic acid (EET) regioisomers. The observed 2- to 3-fold increase in hydrolytic activity over background is, at best, an extremely slow turnover, given that the amount of enzyme used for the assay (100 µg in a 100-µl reaction mixture, corresponding to approximately 2 nmol) was in excess of the amount of the substrate (1.2 nmol) and that the incubation time was 1.5 h.

α/β hydrolase fold enzymes act by a two-step mechanism, with the initial formation of a covalent bond between enzyme and substrate, usually an ester bond (4) (Fig. 1B). If an epoxide hydrolase had a serine instead of an aspartic acid as the catalytic nucleophile, the resulting intermediate, if formed at all, would be an ether rather than an ester (Fig. 1C). Because of the much higher hydrolytic stability of the ether bond than of the ester bond, it is highly unlikely that the second catalytic step that is based on simple water activation of a hydroxy anion can take place with appreciable efficacy.

Taken together, neither the above theoretical considerations nor the experimental data presented by Spillman et al. support the identity of PfEH1 and -2 as epoxide hydrolases. The other observations reported by them, on the other hand, appear to be solid and well founded and suggest a significant role for these enzymes in the EET prevalence in erythrocyte phospholipids. We propose that this is likely due to an esterase activity of the above enzymes.
